# Rab1A mediates proinsulin to insulin conversion in β-cells by maintaining Golgi stability through interactions with golgin-84

**DOI:** 10.1007/s13238-016-0298-x

**Published:** 2016-08-09

**Authors:** Xiaojing Liu, Zhenguo Wang, Ying Yang, Qingrun Li, Rong Zeng, Jiuhong Kang, Jiarui Wu

**Affiliations:** 1 Key Laboratory of Systems Biology, Institute of Biochemistry and Cell Biology, Shanghai Institutes for Biological Sciences, Chinese Academy of Sciences, Shanghai 200031, China; 2 Clinical and Translational Research Center of Shanghai First Maternity and Infant Health Hospital, Shanghai Key Laboratory of Signaling and Disease Research, School of Life Science and Technology, Tongji University, Shanghai, 200092 China; 3 School of Life Science and Technology, ShanghaiTech University, Shanghai, 200031 China; 4 Shanghai Advanced Research Institute, Chinese Academy of Sciences, Shanghai, 201210 China

**Dear Editor,**

Insulin plays a critical role in mammalian glucose homeostasis, and dysfunctional insulin secretion results in diabetes. Insulin maturation in pancreatic β cells can be generally divided into three stages: the first stage is the biosynthesis of proinsulin in the endoplasmic reticulum (ER), the second stage is the transport of proinsulin from the ER into the Golgi, and the third stage is the cleavage of proinsulin into insulin and C peptide in immature insulin granules (Dodson and Steiner, 1998).

Previous studies reported that Rab family, which belongs to the small GTPase family, is involved in the regulation of insulin maturation. For example, Rab3, Rab11, Rab27, and Rab37 were associated with insulin-containing secretory granules and regulation of their exocytosis (Yi et al., 2002; Sugawara et al., 2009; Ljubicic et al., 2013; Cazares et al., 2014). A recent report showed that Rab2A can promote either insulin secretion or ER-associated degradation of proinsulin (Sugawara et al., 2014).

By re-analyzing a dataset of microarray data derived from islets of type 2 diabetes patients (Dominguez et al., 2011), we found that *rab1a* mRNA expression was significantly decreased (*P* < 0.001, Fig. S1A). To confirm this observation, we compared the Rab1A protein levels in islets isolated from Wistar rats with those from diabetic Goto-Kakizaki (GK) rats that have spontaneous type 2 diabetes mellitus. Western blotting analysis showed that Rab1A expression was significantly reduced in GK islets (Fig. S1B). Taken together, Rab1A expression is down-regulated in diabetic islets, implying that Rab1A has an important function in pancreatic β-cells.

We examined the sub-cellular localization of exogenous Rab1A in rat insulinoma INS-1E cells by using confocal microscopy. It has been reported that the Rab1A is mainly localized at the endoplasmic reticulum (ER)-Golgi membranes (Allan et al., 2000; Moyer et al., 2001). In agreement with previous studies, we found that Rab1A is mainly located in the *cis*-Golgi and endoplasmic reticulum (ER)-Golgi intermediate compartment (ER-GIC) in INS-1E cells (Fig. S2). To investigate the function of *rab1a* gene, we used the CRISPR/Cas9 approach to delete the *rab1a* gene in INS-1E cells. Two cell clones (KO-2 and KO-3) were identified that contained additional T nucleotides in the *rab1a* gene, which led to a frameshift mutation (Fig. S3A). No detectable Rab1A was found in KO-2 or KO-3 cells by Western blotting (Fig. S3B) and RT-PCR (Fig. S3C).

We examined glucose-stimulated insulin secretion (GSIS) in INS-1E cells with or without Rab1A function. The results showed that insulin secretion in KO-2 and KO-3 cells was significantly decreased compared with that in the control cells (Fig. 1A). Since the RT-PCR results showed no significant changes in *ins* mRNA expression levels in KO-2 cells (Fig. 1B), we propose that Rab1A is not involved in regulating *ins* transcription. Western-blotting results showed that the proinsulin content of KO-2 cells was not significantly different from that of the control cells, but the matured insulin in KO-2 cells was largely decreased compared with that of the control cells (Fig. 1C). In addition, using rat insulin and proinsulin ELISA kits, we showed that *rab1a* knockout resulted in a significantly decreased insulin content (Fig. 1D, right panel), whereas no significant proinsulin content changes were detected (Fig. 1D, left panel).

**Figure 1:**
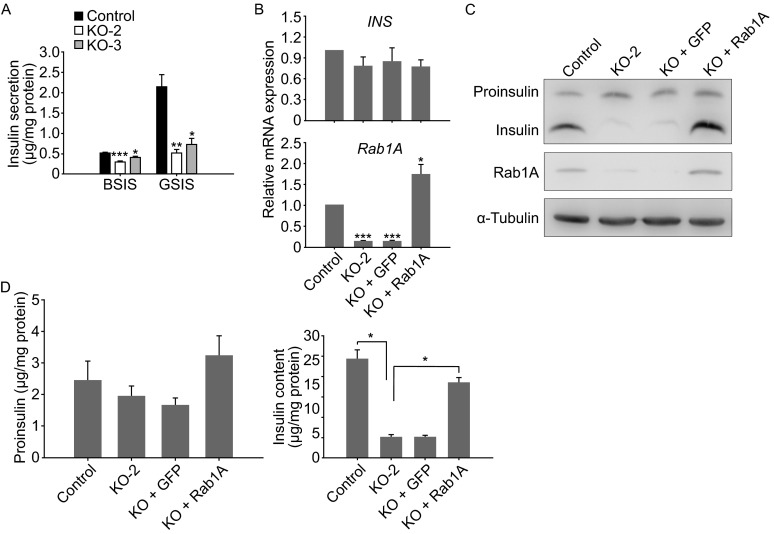
**Insulin content is decreased in** ***rab1a*** **knockout INS-1E cells**. (A) Insulin secretion was detected in INS-1E cells (Control) and *rab1a* knockout cells (KO-2 and KO-3). Insulin secretion levels were measured using a rat insulin ELISA kit. (B) Measurements of *ins* and *rab1a* mRNA expression levels in INS-1E cells (Control), *rab1a* knockout cells (KO-2 and KO + GFP) and exogenous *rab1a* expression cells (KO + Rab1A) by RT-PCR. (C) Measurements of proinsulin and insulin contents in the cells as described in Fig. 1B by Western blotting. (D) Measurements of proinsulin and insulin contents in the cells as described in Fig. 1B using the rat proinsulin ELISA kit (left panel) and insulin ELISA kit (right panel), respectively. The results of (A) and (C and D) are presented as the mean ± S.E.M. (*n* = 3). *, *P* < 0.05; **, *P* < 0.01; ***, *P* < 0.001

We next expressed wild type Rab1A and its mutants Q70L (a GTP-restricted mutant, activated form) and S25N (a GDP-restricted mutant, inactivated form) by lentivirus infection to see whether the activated form of Rab1A is essential to INS-1E cells’ function. We observed that overexpression of Rab1A WT or Rab1A Q70L resulted in no apparent increase of basal insulin secretion (BSIS), GSIS and insulin content. This lack of effect was not surprising, given that endogenous activated form of Rab1A may be functionally enough. But overexpression of Rab1A S25N significantly reduced BSIS, GSIS and insulin content (Fig. S4), suggesting that the activated form of Rab1A is essential to insulin secretion and insulin content.

To further confirm that Rab1A regulates proinsulin to insulin conversion, we established KO + Rab1A cells by infecting KO-2 cells with a Rab1A-lentivirus (Fig. 1B and 1C). The exogenous expression of Rab1A in KO + Rab1A cells recovered the insulin content to levels that were similar to those observed in the control INS-1E cells (Fig. 1C and right panel of 1D), whereas no significant increase in proinsulin content was detected in these KO + Rab1A cells (Fig. 1C and left panel of 1D). These results indicate that exogenous Rab1A expression could rescue decreased insulin content in Rab1A-knockout cells. Taken together, we concluded that Rab1A plays an important role in the conversion of proinsulin to insulin.

Rab1 has two isoforms, Rab1A and Rab1B, which share 92% amino acid identity (Touchot et al., 1989). The present results showed that the *rab1a* gene was successfully knocked out in KO-2 and KO-3 cells without influencing Rab1B protein expression (Fig. S3B). To detect whether Rab1B plays the similar role as Rab1A, we transfected small interfering RNA (siRNA) to knockdown *rab1b* in INS-1E cells (Fig. S5A). The results showed that insulin content in the *rab1b* knockdown cells was the same as that in the control cells (Fig. S5B), indicating that Rab1B is not involved in proinsulin to insulin conversion.

Using electron microscopy, we evaluated the ultrastructure of Golgi in the cells. The results showed that Golgi ribbon sizes were significantly reduced in *rab1a* knockout cells (KO-2, KO + GFP, Fig. 2A and B). Further analysis of Golgi cisternae indicated that large Golgi cisternae (>0.05 μm^2^) numbers were significantly decreased in *rab1a* knockout cells (Fig. 2C), whereas small Golgi cisternae (0.002–0.01 μm^2^) numbers were significantly increased (Fig. 2C). Importantly, exogenous Rab1A expression in *rab1a* knockout cells (KO + Rab1A) restored the Golgi ribbon structures to resemble those of the control cells (Fig. 2A–C), suggesting that Rab1A is required for Golgi stability.

**Figure 2:**
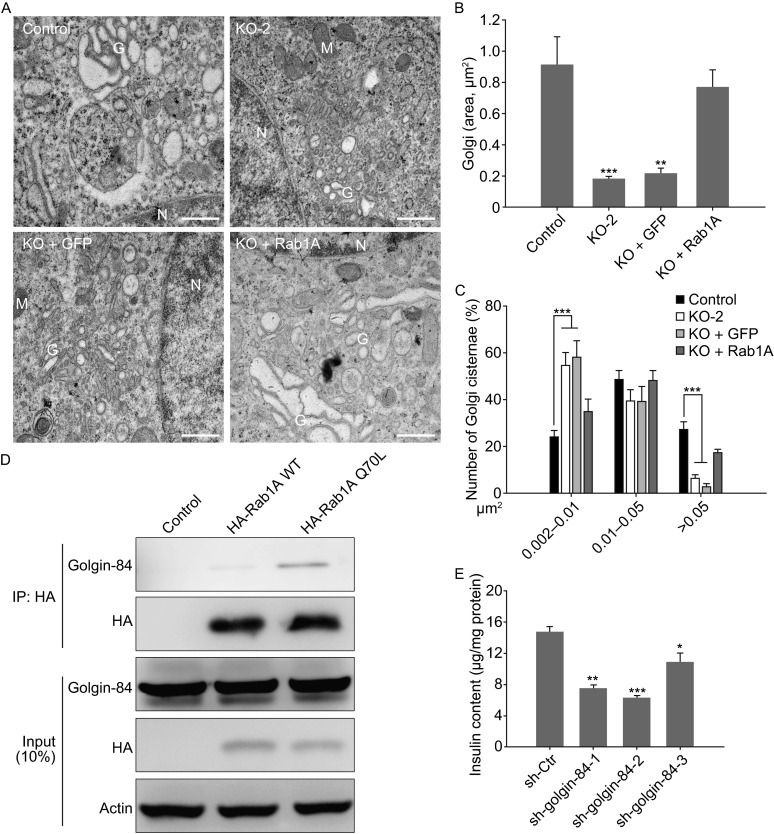
***Rab1a*** **knockout results in Golgi ribbon fragmentation**. (A) Electron micrograph of the cells as described in Fig. 1B (G, Golgi; M, mitochondria; N, nucleus). All images are presented at 21,000× magnification. Scale bars, 0.5 μm. (B and C) Golgi (B) and Golgi cisternae (C) areas in the electron micrographic images were measured using Image Pro Plus (numbers of measured cells: Control, *n* = 21; KO-2, *n* = 20; KO + GFP, *n* = 14; KO + Rab1A, *n* = 14). (D) Immunoprecipitation of Rab1A interaction proteins by anti-HA antibody and analyzed with anti-golgin-84 antibody by Western blotting. IP: immunoprecipitation. (E) Insulin content in *golgin-84* shRNAs infected INS-1E cells was determined. The results are presented as the mean ± S.E.M. (*n* = 3). *, *P* < 0.05; **, *P* < 0.01; *** *P* < 0.001

Since a recent report showed that Rab2A is involved in the regulation of ER stress within insulin-secreting cells (Sugawara et al., 2014), we wondered whether *rab1a* knockout would increase ER stress in INS-1E cells. Rough ER has normal flattened cisternae structure that is packed with polyribosomes. Dramatic ER distension is usually an indicative of severe ER stress (Wikstrom et al., 2013). We used electron microscopy to evaluate ultrastructure of ER in the cells. No obvious difference of ER morphology was observed between Ctr and *rab1a* knockout cells (Fig. S6A, compare KO-2 and KO + GFP with Ctr). The rough ER in the *rab1a* knockout cells have normal flattened cisternae structure, which are packed with polyribosomes. We also detected several ER-stress marker protein expression levels by Western bloting. Western blotting results showed that several ER-stress marker protein expression levels in *rab1a* knockout cells were similar to those of the control cells (Fig. S6B), suggesting that *rab1a* knockout does not induce ER stress in insulin-secreting cells. Because we did not observe proinsulin accumulation in *rab1a* knockout cells (Fig. 1C, left panel of 1D), we proposed that there may be a negative feedback loop regulating proinsulin production in insulin-secreting cells that may prevent proinsulin accumulation when the proinsulin conversion to insulin is impeded to avoid ER stress.

Since a number of studies showed that Rab GTPases carry out their functions through the recruitment of various effector proteins (Allan et al., 2000; Sugawara et al., 2009; Ljubicic et al., 2013; Sugawara et al., 2014), we used mass spectrometry to identify Rab1A effectors that can maintain Golgi stability. Using *rab1a* knockout cells, we constructed cell lines that overexpressed HA-tagged wild-type (WT) or constitutively active (Q70L) Rab1A (Short et al., 2001). Then, both HA-tagged Rab1A WT and Q70L were immunoprecipitated and then subjected to mass spectrometry. We identified a total of 447 proteins, among which 18 were detected in both Rab1A-WT and -Q70L samples but not in Rab1A knockout samples (Table S2), suggesting that these 18 proteins were likely to interact with Rab1A.

Previous studies showed that a Golgi membrane protein golgin-84, which is shown in Table S2, can interact with Rab1 to maintain Golgi structure (Diao et al., 2003; Satoh et al., 2003). Therefore, we speculated that golgin-84 interacts with Rab1A in INS-1E cells. To confirm this observation, we immunoprecipitated golgin-84 with anti-HA antibody. The result showed that golgin-84 was detected in both Rab1A-WT and -Q70L samples, but not in Rab1A knockout samples (Fig. 2D). Furthermore, the expression of golgin-84 in the Rab1A-Q70L sample was higher than that in the Rab1A-WT sample (Fig. 2D), implying that the constitutively active Rab1A-Q70L cells may generate stronger golgin-84-binding activity. We also found that exogenously expressed EGFP-Rab1A colocalized with golgin-84 in INS-1E cells (Fig. S7A). Taken together, Rab1A interacts with golgin-84 in INS-1E cells.

Approximately 20 of the 70 known Rab proteins are associated with the Golgi apparatus (Liu and Storrie, 2015). In a recent review paper, Liu and Storrie proposed that there are two classes of Golgi-associated Rab proteins: in Class 1, Rab inactivation leads to Golgi ribbon disruption; in Class 2, Rab inactivation has little to no obvious effect on Golgi organization (Liu and Storrie, 2015). However, both Rab1A and Rab1B are categorized as Class 1 Rab proteins (Table 2 in reference (Liu and Storrie, 2015)). The present result that *rab1a* depletion disrupts Golgi ribbon organization (Fig. 2A–C) supports this hypothesis. Identification of golgin-84 as a Rab1A-associating protein (Fig. 2D) in our present study further suggests that this type of Golgi ribbon fragmentation may result from the loss of Rab1A and golgin-84 interactions.

Based on the observation that *rab1a* knockout resulted in inhibition of the proinsulin to insulin conversion (Fig. 1C and 1D), we proposed that down-regulation of *golgin-84* expression may also impede proinsulin to insulin conversion. Therefore, we knocked down *golgin-84* expression using shRNAs in INS-1E cells (Fig. S7B and S7C) and observed that the insulin content decreased as we expected (Fig. 2E). But interestingly, knockdown of golgin-84 in INS-1E cells also resulted in decreased proinsulin content (Fig. S7D). This may because that knockdown of golgin-84 not only disrupts Rab1A and golgin-84 interaction but also influences other functions of golgin-84, which may need further studies. Furthermore, because *golgin-84* depletion also induced Golgi ribbon fragmentation (Diao et al., 2003; Satoh et al., 2003), we conclude that the functional interaction between Rab1A and golgi-84 maintaining Golgi stability is critical to proinsulin to insulin conversion.

In conclusion, our findings indicate that Rab1A interacts with golgin-84 to maintain the Golgi ribbon structure and required for converting proinsulin to insulin within insulin-secreting cells. The disruption of the interaction between Rab1A and golgin-84 results in inhibition of the proinsulin to insulin conversion due to Golgi ribbon fragmentation.

## Footnotes

This work was supported by grants to Wu JR from the National Natural Science Foundation of China (Grant Nos. 31130034 and 31470808), and Strategic Priority Research Program of the Chinese Academy of Sciences (XDA12000000).

Xiaojing Liu, Zhenguo Wang, Ying Yang, Qingrun Li, Rong Zeng, Jiuhong Kang, and Jiarui Wu declare that they have no conflict of interest. All institutional and national guidelines for the care and use of laboratory animals were followed.

## Electronic supplementary material

Below is the link to the electronic supplementary material.

Supplementary material 1 (PDF 8465 kb)

## References

[CR1] Allan BB, Moyer BD, Balch WE (2000). Rab1 recruitment of p115 into a cis-SNARE complex: programming budding COPII vesicles for fusion. Science.

[CR2] Cazares VA, Subramani A, Saldate JJ, Hoerauf W, Stuenkel EL (2014). Distinct actions of Rab3 and Rab27 GTPases on late stages of exocytosis of insulin. Traffic.

[CR3] Diao A, Rahman D, Pappin DJ, Lucocq J, Lowe M (2003). The coiled-coil membrane protein golgin-84 is a novel rab effector required for Golgi ribbon formation. J Cell Biol.

[CR4] Dodson G, Steiner D (1998). The role of assembly in insulin’s biosynthesis. Curr Opin Struct Biol.

[CR5] Dominguez V, Raimondi C, Somanath S, Bugliani M, Loder MK, Edling CE, Divecha N, da Silva-Xavier G, Marselli L, Persaud SJ (2011). Class II phosphoinositide 3-kinase regulates exocytosis of insulin granules in pancreatic beta cells. J Biol Chem.

[CR6] Liu S, Storrie B (2015). How Rab proteins determine Golgi structure. Int Rev Cell Mol Biol.

[CR7] Ljubicic S, Bezzi P, Brajkovic S, Nesca V, Guay C, Ohbayashi N, Fukuda M, Abderrhamani A, Regazzi R (2013). The GTPase Rab37 Participates in the Control of Insulin Exocytosis. PLoS ONE.

[CR8] Moyer BD, Allan BB, Balch WE (2001). Rab1 interaction with a GM130 effector complex regulates COPII vesicle cis-Golgi tethering. Traffic.

[CR9] Satoh A, Wang Y, Malsam J, Beard MB, Warren G (2003). Golgin-84 is a rab1 binding partner involved in Golgi structure. Traffic.

[CR10] Short B, Preisinger C, Korner R, Kopajtich R, Byron O, Barr FA (2001). A GRASP55-rab2 effector complex linking Golgi structure to membrane traffic. J Cell Biol.

[CR11] Sugawara K, Shibasaki T, Mizoguchi A, Saito T, Seino S (2009). Rab11 and its effector Rip11 participate in regulation of insulin granule exocytosis. Genes Cells.

[CR12] Sugawara T, Kano F, Murata M (2014). Rab2A is a pivotal switch protein that promotes either secretion or ER-associated degradation of (pro)insulin in insulin-secreting cells. Sci Rep.

[CR13] Touchot N, Zahraoui A, Vielh E, Tavitian A (1989). Biochemical properties of the YPT-related rab1B protein. Comparison with rab1A. FEBS Lett.

[CR14] Wikstrom JD, Israeli T, Bachar-Wikstrom E, Swisa A, Ariav Y, Waiss M, Kaganovich D, Dor Y, Cerasi E, Leibowitz G (2013). AMPK regulates ER morphology and function in stressed pancreatic beta-cells via phosphorylation of DRP1. Mol Endocrinol.

[CR15] Yi Z, Yokota H, Torii S, Aoki T, Hosaka M, Zhao S, Takata K, Takeuchi T, Izumi T (2002). The Rab27a/granuphilin complex regulates the exocytosis of insulin-containing dense-core granules. Mol Cell Biol.

