# Supplementary material for: Rab1A mediates proinsulin to insulin conversion in β-cells by maintaining Golgi stability through interactions with golgin-84
Source: Protein Cell. 2016 Aug 9;7(9):692–6. doi: 10.1007/s13238-016-0298-x (PMC5003788; doi:10.1007/s13238-016-0298-x)
Supplement: Supplementary file 1 — Supplementary material 1 (PDF 8465 kb) [file 13238_2016_298_MOESM1_ESM.pdf]

Supplementary materials

MATERIALS AND METHODS

Animals

Male Wistar and GK rats aged 6-8 weeks were purchased from the Shanghai Laboratory Animal Center. All animals were maintained in accordance with the guidelines of the Institutional Animal Care and Use Committee of the Institute of Biochemistry and Cell Biology, Chinese Academy of Sciences.

Cell culture

Rat insulinoma INS-1E cells were cultured as previously described (Merglen et al., 2004), and HEK-293 cells (human embryonic kidney cells) were maintained in DMEM (Thermo Fisher Scientific, Carlsbad, CA, United States) supplemented with 10% fetal bovine serum (Thermo Fisher Scientific, Carlsbad, CA, United States) at 37 °C and 5% CO2.

Plasmids

The rat *rab1a* coding region was inserted into the pCDH-CMV-MCS-EF1-Puro vector. Rab1A Q70L and Rab1A S25N were generated using site-directed mutagenesis PCR (QuikChange Lightning Site-Directed Mutagenesis Kit, Agilent Technologies, Santa Clara, CA, United States). As for pEGFP-Rab1A plasmid, the rat *rab1a* coding region was inserted into the pEGFP-C1 vector (Sannerud et al., 2006).

Immunofluorescence Staining and Confocal Microscopy

The INS-1E cells were transfected with pEGFP-Rab1A expression vector using Lipofectamine 3000 (Thermo Fisher Scientific, Carlsbad, CA, United States). After 24 hours, cells were fixed in 4% paraformaldehyde, perforated in 0.2% Triton-X-100 and blocked in 1% BSA. The cells were then incubated for 1 hour with the following primary antibodies: GM130 (610822, BD Biosciences, New Jersy, United States, 1:500), p58 (13364, Proteintech Group, Chicago, IL, United States, 1:100) or golgin-84 (HPA000992, Sigma-Aldrich, St. Louis, MO, United States, 1:100). The cells were then incubated for 1 hour Cy3-conjugated goat-anti-rabbit or goat-anti-mouse secondary antibodies (Molecular Probes, Thermo Fisher Scientific, Carlsbad, CA, United States), then co-labeled with DAPI (Sigma, St. Louis, MO, United States). Images were acquired using a Leica TCS SP5 confocal microscope.

***Rab1a* knockout in INS-1E Cells using the CRISPR/Cas9 approach**

Using the CRISPR Design Tool (http://tools.genome-engineering.org), we designed single-guide RNA (sgRNA) and cloned the sgRNA into the pX330-U6-Chimeric_BB-CBh-hSpCas9 vector, according to a previous protocol (Ran et al., 2013). The following 20-nt *rab1a* sgRNA sequence was used: 5’-CAACCCCAGAGTCGCCAATC-3’, directly upstream of a 5’-AGG protospacer adjacent motif (PAM).

The INS-1E cells were transfected with 12 μg of pX330 *rab1a* sgRNA expression vector using Lipofectamine 2000 (Thermo Fisher Scientific, Carlsbad, CA, United States). Single cells were isolated in 96-well plates using a BD AriaII sorter gated on mCherry fluorescence. Genomic DNA samples were extracted using a DNeasy Blood

and Tissue Kit (Qiagen, Hilden, Germany) according to the manufacturer's protocol. PCR amplification was performed using genomic DNA as a template, and the PCR products were sequenced and analyzed.

Rab1a overexpression in INS-1E Cells

HEK-293T cells were co-transfected with psPAX2 and pMD2.G lentivirus packaging vectors with pCDH-GFP, Rab1A WT, Rab1A Q70L or Rab1A S25N using Lipofectamine 2000 (Thermo Fisher Scientific, Carlsbad, CA, United States), according to the manufacturer's protocol. After a 48-hour transfection, the lentivirus particles were collected from the HEK-293T cells and transfected into INS-1E cells. After a 48-hour infection, these INS-1E cells were used for insulin secretion and insulin content measurements.

siRNA knockdown of _rab1b_

Three siRNAs against rat rab1b (NCBI Gene ID: 100126191) were synthesized by Shanghai GenePharma Co., Ltd.（Suzhou, Jiangsu, China）. The target sequences were used: 5'-TGTTTCACATTAGCGTAGG-3', 5'-TCATACACCACAATGATGC-3' and 5'-TTCGAATCTTGAAGTCCAC-3'. The three siRNAs pool was used to knockdown rab1b. The siRNA (5'-TTCTCCGAACGTGTCACGT-3') was used as the control siRNA, which does not target any sequence present in the rat genome.

INS-1E cells were transfected with 50 nM rab1b siRNAs pool using Lipofectamine 3000 (Thermo Fisher Scientific, Carlsbad, CA, United States) according to the manufacturer's protocol. After a 72-hour transfection, the INS-1E were subjected to Western-blotting or insulin content measurement.

Insulin secretion and insulin content detection

To measure glucose-stimulated insulin secretion (GSIS), INS-1E cells were pre-incubated for 1 hour at 37 °C in Krebs-Ringer bicarbonate buffer (KRBH) (Han et al., 2011) and then incubated with 2.8 mM or 16.7 mM glucose for 1 hour. The insulin in the media was measured using a rat insulin ELISA kit (Mercodia, Uppsala, Sweden). In addition, these cells were lysed using cell lysis buffer (Beyotime Biotechnology, Haimen, Jiangsu, China), and total cellular insulin (insulin content) was extracted using an ethanol-water-HCl solution (52:17:1 v/v) and measured using the insulin ELISA kit (Mercodia, Uppsala, Sweden).

Quantitative real-time PCR

Total RNA was extracted using TRIZOL Reagent (Thermo Fisher Scientific, Carlsbad, CA, United States), according to the manufacturer's protocol from INS-1E cells. One microgram of mRNA isolated from INS-1E cells was used for reverse transcription. The primers used are listed in Table S1. The cycling procedure used for real-time PCR (CFX96 Touch, Bio-Rad Laboratories, United States) for all of the samples was as follows: an initial step at 95 °C for 5 minutes, followed by 40 cycles of 95 °C for 10 seconds, 60 °C for 20 seconds and 72 °C for 20 seconds. The expression levels of *rab1a* and *ins* mRNA were normalized to *β-actin* mRNA levels.

Lentiviral infection and co-immunoprecipitation

HEK-293T cells were co-transfected with psPAX2 and pMD2.G lentivirus packaging vectors with pCDH HA-Rab1A WT or HA-Rab1A Q70L using Lipofectamine 2000

(Thermo Fisher Scientific, Carlsbad, CA, United States), according to the manufacturer's protocol. After a 48-hour transfection, the lentivirus particles were collected from the HEK-293T cells and transfected into INS-1E cells. After a 48-hour infection, these INS-1E cells were used for immunoprecipitation. INS-1E cells were lysed in IP buffer (150 mM NaCl, 50 mM Tris, 5 mM EDTA, 1% (v/v) NP-40, and Protease Inhibitor Cocktail (Sigma-Aldrich, USA)). The cell lysate was rotated for 30 minutes and centrifuged at 13,300 rpm at 4 °C for 15 minutes. The supernatant was incubated with anti-HA-conjugated beads (M20013, Abmart, China) at 4 °C for 2 hours. Proteins were eluted by glycine (0.2 M, pH 2.5) and adjusted to pH 7.5 with 1.5 M Tris, before Western-blotting or mass spectrometry analysis.

Western blotting

Proteins were separated on Laemmli-SDS-PAGE or Tricine-SDS-PAGE, transferred to PVDF membranes and detected with primary antibodies as follows: Rab1A (11671, Proteintech Group, Chicago, IL, United States, 1:1000), Rab1B (17824, Proteintech Group, Chicago, IL, United States, 1:1000), Actin (sc-1616, Santa Cruz biotechnology, Inc., Dallas, Texas, United States, 1:2000), α-tubulin (T6199, Sigma-Aldrich, USA, 1:5000), insulin (I2018, Sigma-Aldrich, St. Louis, MO, United States, 1:1000) and golgin-84 (HPA000992, Sigma-Aldrich, St. Louis, MO, United States, 1:1000); Bip, pIRE1α and IRE1α were gifts from Dr. Yong Liu (Institute for Nutritional Sciences, CAS, 1:1000).

Electron microscopy analysis

Transmission electron microscopy (TEM) analysis was performed as follows: INS-1E cells were trypsinized, fixed in 2.5% glutaraldehyde in ice-cold PBS for 24 hours, and then fixed with 2% OsO_4 in ice-cold PBS for 1.5 hours. The fixed cells were dehydrated in a graded series of ethanol (30%, 50%, 70%, 80%, 95%, and 100%) for 10 minutes at each grade. The samples were embedded in Epon 812 resin, and ultrathin sections of 70 nm were made. The ultrathin sections were contrasted with 2% uranyl acetate and lead citrate. The samples were viewed with a Tecnai G2 Spirit TEM (FEI, USA). The Golgi areas were analyzed using Image-Pro Plus 6.0 software (Media Cybernetics, Silver Spring, Maryland, United States).

LC-MS analysis and data acquisition

Proteins were immunoprecipitated with anti-HA-conjugated agarose and eluted with glycine. The eluted proteins were adjusted to pH 7.5 with 1.5 M Tris, reduced with 0.5 mM DTT for 30 minutes, alkylated with 3 mM iodoacetamide at room temperature for 30 minutes in the dark, digested with trypsin and incubated overnight at 37 °C. Peptides were extracted in 0.2% trifluoroacetic acid in 80% acetonitrile and vacuum dried. Liquid chromatography separation was performed on the high-pressure nanoflow HPLC Easy nLC 1000 system (ThermoFisher Scientific, USA). The lab-made column (15 cm in length, 75 μm inner diameter) was packed with ReproSil-Pur C18-AQ 3 μm resin (Dr. Maisch GmbH) in 100% methanol. For high mass accuracy data acquisition, a Q-Exactive mass spectrometer (ThermoFisher Scientific, USA) was used and equipped with a nanoelectrospray ion source (ThermoFisher Scientific, USA). Peptides were loaded onto the column and separated

with a linear gradient of 5-35% buffer B (ACN with 0.1% formic acid) at a flow rate of 300 nL/min over 40 min.

Data were acquired in the data-dependent "top12" mode, in which the 12 most abundant precursor ions were selected with high resolution (70,000 @ m/z 200) from the full scan (300-1,800 m/z) for HCD fragmentation. Precursor ions with singly charged or unassigned charge information were excluded. The resolution for the MS/MS spectra was set to 17,500 @ m/z 200, the target value was 1E5 (AGC control enabled), and the isolation window was set to 2.0 m/z. The normalized collision energy was 30.

Database search

All mass spectrometric data were analyzed against the uniprot rat database (201307, 28894 proteins) using MaxQuant 1.5.2.8 (Cox et al., 2014). Enzyme specificity was set to trypsin/P. Two missing cleavage sites were allowed. For MS and MS/MS, the tolerance levels of the main peptide search were set at 7 ppm and 20 ppm, respectively. The peptide and protein false discovery rate (FDR) was fixed at a significant level not greater than 0.01. Protein raw intensity and label free quantitation (LFQ) values were calculated using MaxQuant, as previously described (Cox et al., 2014).

Knocking down *golgin-84* expression with shRNAs

Targeted sequences against rat golgin-84 (NCBI Gene ID: 299258) were cloned into pLKO.1-TRC-EGFP vectors. The sequences used were as follows:

sh-golgin-84-1: GCAAGAGAGCTACAAACAAAT;

sh-golgin-84-2: GGACCGAATGAAACAGGAATT;

sh-golgin-84-3: AATTTAGTATTCGCCTGGGAA.

HEK-293T cells were co-transfected with psPAX2 and pMD2.G lentivirus packaging vectors with pLKO.1-TRC-EGFP shRNA encoding plasmids using Lipofectamine 2000 (Thermo Fisher Scientific, USA), according to the manufacturer's protocol. After a 48-hour transfection, the lentivirus particles were collected from the HEK-293T cells and transfected into INS-1E cells. After a 4-day infection, the INS-1E cells were subjected to Western-blotting or insulin content measurement.

Statistical analysis

Data were obtained from at least three independent experiments and are presented as the mean ± standard error of the mean (S.E.M.). Statistical significance between the two samples was assessed using Student's t-test. $P<0.05$ was considered significant.

Table S1. qPCR primers used in this study.

rab1a-Forward	5'-GGATTTCAAGATACGGACT-3'
rab1a-Reverse	5'- ACATTTGTTCCCTACCA-3'
ins-Forward (Kim et al., 2016)	5'-TGCCCAGGCTTTTGTCAAACAGCACCTT-3'
ins-Reverse (Kim et al., 2016)	5'-CTCCAGTGCCAAGGTCTGAA-3'
β-actin-Forward (Kim et al., 2015)	5'-GTCGTACCACTGGCATTGTG-3'
β-actin-Reverse (Kim et al., 2015)	5'-CTCTCAGCT GTGGTGGTGAA3'

Table S2. Putative Rab1A-interacting proteins identified by mass spectrometer

**Protein names**	**MS/MS Counts**
[a,b]Rab GDP dissociation inhibitor alpha	66
[a,b]Peroxiredoxin-1	14
[a,b]V-type proton ATPase 116 kDa subunit a isoform 1	14
[a,b]Atp6v1h	1
[a,b]Golgin-84	3
[a]Guanine nucleotide exchange factor MSS4	1
[b]Phosphoglycerate mutase 2	1
[b]Tfrc	1
[b]Peroxiredoxin-1	14
[b]Alpha-crystallin A chain	3
[b]Cofilin-1	3
[b]ATP-dependent RNA helicase SUPV3L1	1
[b]Lumican	1
[b]Decorin	3
[b]Cellular retinoic acid-binding protein 1	4
[b]Tubulin beta-2B	1
[b]L-lactate dehydrogenase	3
[b]Proteasome subunit beta type-6	8

[a] indicates proteins detected in the Rab1A WT sample but not in the control sample;

[b] indicates proteins detected in the Rab1A Q70L sample but not in the control sample.

SUPPLEMENTARY FIGURE LEGENDS

Fig. S1. Rab1A expression is decreased in diabetic islets. (A) *Rab1a* mRNA expression in human islets was analyzed from previous microarray data of seven non-diabetic individuals and six type 2 diabetes patients (Dominguez et al., 2011). The values are presented as the mean ± S.E.M. ***, P<0.001. (B) Rab1A protein expression in Wistar and GK rat islets (n = 3) was detected by Western blotting (left panel). Rab1A protein expression levels were calculated and normalized to Actin (right panel). The values are presented as the mean ± S.E.M. **, P<0.01.

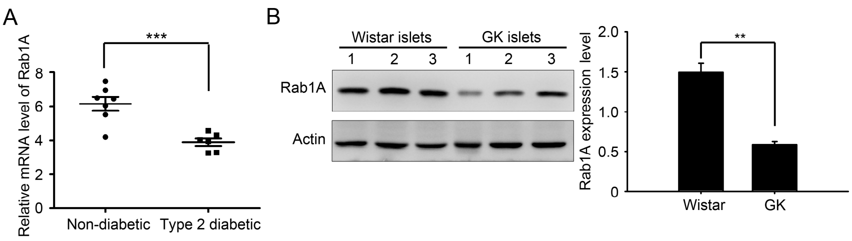

Fig. S2. Cellular localization of Rab1A in INS-1E cells. (A-B) INS-1E cells transiently expressing EGFP-Rab1A were stained with antibodies against GM130 (*cis*-Golgi) (A) and p58 (ER-GIC) (B). The images were analyzed by confocal microscopy. Representative images are shown. White arrows indicate the colocalization of EGFP-Rab1A with GM130 or p58. Scale bars, 7.5 µm.

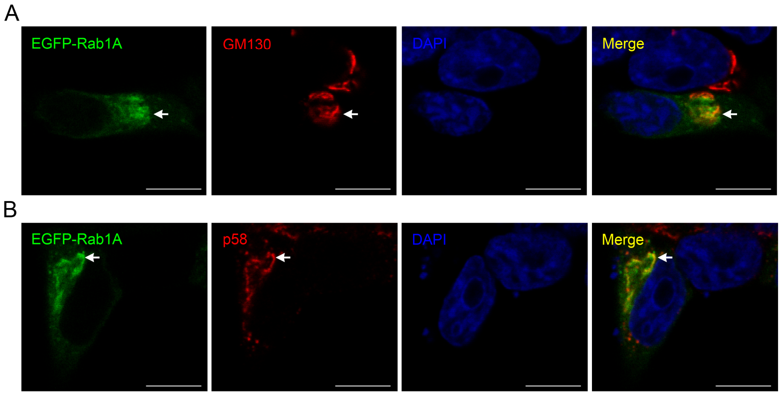

Fig. S3. Efficiency of *rab1a* kncokout in INS-1E cells. (A) The cell lines KO-2 and KO-3 contained a additional T nucleotides in the *rab1a* gene in INS-1E cells and led to frameshift mutation, which resulted in *rab1a* knockout. (B) The expression levels of Rab1A and Rab1B were detected in the control, KO-2 and KO-3 cells by Western blotting. (C) The *rab1a* mRNA expression levels from the control, KO-2 and KO-3 cells were measured by RT-PCR. The *rab1a* mRNA expression was normalized to that of the control cells. The results are presented as the mean ± S.E.M. (n = 3). ***, *P*<0.001.

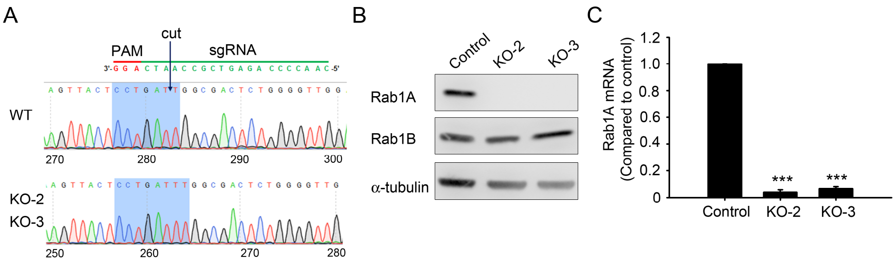

Fig. S4. Insulin secretion and insulin content are decreased in inactivated form of Rab1A overexpressing INS-1E cells. (A) Insulin secretion was detected in INS-1E cells transiently expressing GFP, wildtype Rab1A （Rab1A WT）, activated form of Rab1A (Rab1A Q70L) or inactivated form of Rab1A (Rab1A S25N). Insulin secretion levels were measured using a rat insulin ELISA kit. (B) Measurements of insulin contents in the cells as described in Fig. S4A. The results are presented as the mean ± S.E.M. (n = 3). *, *P*<0.05; **, *P*<0.01. Rab1A S25N versus GFP.

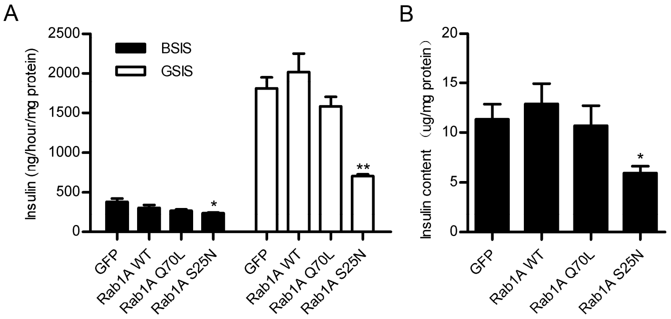

Fig. S5. *Rab1b* knockdown has no effect on insulin content in INS-1E cells. (A) The INS-1E cells were transfected with 50 nM *rab1b* siRNAs pool or control siRNA for 72 hours. Rab1B expression was detected by Western blotting (upper panel) and quantified (lower panel) in the INS-1E cells. The result is presented as the mean ± S.D. (n = 3). **, *P*<0.01; (B) Insulin contents in INS-1E cells, which were transfected with 50 nM *rab1b* siRNAs pool or control siRNA for 72 hours, were measured with rat insulin ELISA kit. The result is presented as the mean ± S.D. (n = 3).

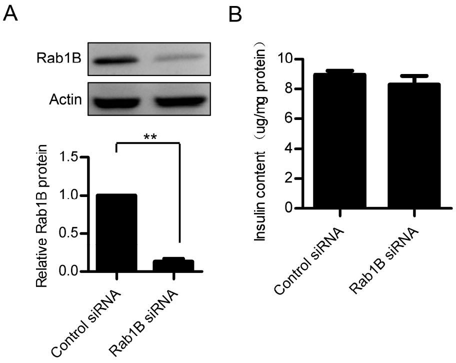

Fig. S6. Effects of the *rab1a* knockout on ER. (A) There was no significant difference on ER morphology in the *rab1a* knockout cells to that of the control cells. Electron micrographs of INS-1E cells (Control), the *rab1a* knockout cells (KO-2 and KO+GFP) and the exogenous expression Rab1A cells (KO+Rab1A) (rough endoplasmic reticulum; M, mitochondria; N, nucleus). All images are presented at 21,000x magnification. Scale bars, 0.5 μm. (B) No significant ER-stress in the *rab1a* knockout cells was detected. Western blotting analyses (upper panel) of ER-stress markers (BiP, IRE1α and pIRE1α) of INS-1E cells (Control), the *rab1a* knockout cells (KO-2 and KO+GFP) and the exogenous expression Rab1A cells (KO+Rab1A). The relative levels of BiP and phosphorylated IRE1α were quantified (lower panel). The results are presented as the mean ± S.E.M. (n = 3).

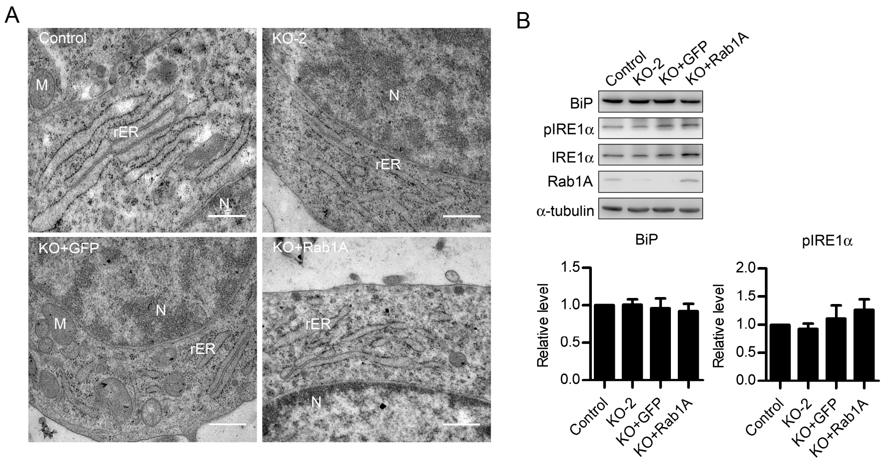

Fig. S7. Efficiency of *golgin-84* knockdown in INS-1E cells. (A) Exogenous EGFP-Rab1A colocalized with golgin-84. INS-1E cells transiently expressing EGFP-Rab1A were stained with antibodies against golgin-84. The images were analyzed by confocal microscopy. Representative images are shown. White arrows

indicate the colocalization of EGFP-Rab1A with golgin-84. Scale bars, 7.5 μm. (B-C) Golgin-84 expression was detected by Western blotting (B) and quantified (C) in INS-1E cells, which were infected with lentiviral *golgin-84* shRNAs. (D) Measurements of proinsulin contents in the *golgin-84* knockdown cells using the rat proinsulin ELISA kit. The results are presented as the mean ± S.E.M. (n = 3). *, *P*<0.05; **, *P*<0.01. Comapred to sh-Ctr.

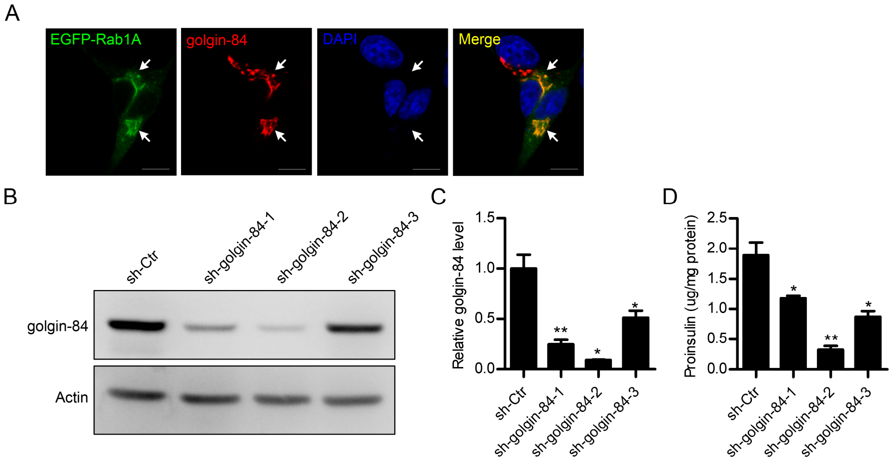

SUPPLEMENTARY REFERENCES

Cox, J., Hein, M.Y., Luber, C.A., Paron, I., Nagaraj, N., and Mann, M. (2014). Accurate proteome-wide label-free quantification by delayed normalization and maximal peptide ratio extraction, termed MaxLFQ. Mol Cell Proteomics 13:2513-2526.

Dominguez, V., Raimondi, C., Somanath, S., Bugliani, M., Loder, M.K., Edling, C.E., Divecha, N., da Silva-Xavier, G., Marselli, L., Persaud, S.J., *et al.* (2011). Class II phosphoinositide 3-kinase regulates exocytosis of insulin granules in pancreatic beta cells. J Biol Chem 286:4216-4225.

Han, S., Pan, H., Zhang, J., Tan, L., Ma, D., Yuan, J., and Wu, J.R. (2011). Identification of a small molecule activator of novel PKCs for promoting glucose-dependent insulin secretion. Cell Res 21:588-599.

Kim, J.K., Lim, Y., Lee, J.O., Lee, Y.S., Won, N.H., Kim, H., and Kim, H.S. (2015). PRMT4 is involved in insulin secretion via the methylation of histone H3 in pancreatic beta cells. J Mol Endocrinol 54:315-324.

Kim, J.W., You, Y.H., Ham, D.S., Yang, H.K., and Yoon, K.H. (2016). The Paradoxical Effects of AMPK on Insulin Gene Expression and Glucose-Induced Insulin Secretion. J Cell Biochem 117:239-246.

Merglen, A., Theander, S., Rubi, B., Chaffard, G., Wollheim, C.B., and Maechler, P. (2004). Glucose sensitivity and metabolism-secretion coupling studied during two-year continuous culture in INS-1E insulinoma cells. Endocrinology 145:667-678.

Ran, F.A., Hsu, P.D., Wright, J., Agarwala, V., Scott, D.A., and Zhang, F. (2013). Genome engineering using the CRISPR-Cas9 system. Nat Protoc 8:2281-2308.

Sannerud, R., Marie, M., Nizak, C., Dale, H.A., Pernet-Gallay, K., Perez, F., Goud, B., and Saraste, J. (2006). Rab1 defines a novel pathway connecting the pre-Golgi intermediate compartment with the cell periphery. Mol Biol Cell 17:1514-1526.
